# Trends in Tattoo-Related Google Search Data in the United States: Time-Series Analysis

**DOI:** 10.2196/40540

**Published:** 2022-12-01

**Authors:** Benjamin Matthew Kiszla, Mia Broughton Harris

**Affiliations:** 1 Heersink School of Medicine University of Alabama at Birmingham Birmingham, AL United States

**Keywords:** big data, dermatoepidemiology, infodemiology, tattoo, United States, web search, dermatology, tattoo care, skin care, guidance seeking, tattoo removal, tattoo application, information seeking, internet search, web searches, adverse reactions

## Abstract

**Background:**

Tattoos are becoming increasingly common in the United States. However, little information is available to help clinicians anticipate where, when, and on what topics patients will seek guidance regarding tattoo care, complications, and removal.

**Objective:**

The aim of this study was to model web searches concerning general interest in tattoo application, tattoo removal, and the geolocation of tattooing services.

**Methods:**

Relative search volumes (RSVs) were elicited from Google Trends, filtered to web searches made in the United States between January 15, 2008, and October 15, 2022. Longitudinal data were analyzed in GraphPad Prism and geospatial data were visualized with Datawrapper for general interest searches (*tattoo* and *tattoo removal*), aggregated geolocating searches (eg, *tattoo shops near me*), and symptomatic searches relating to adverse effects (eg, *itchy tattoo*). Results were compared to previous global literature and national surveys of tattoo prevalence.

**Results:**

In the United States, the search terms *tattoo* and *tattoo removal* have experienced stable RSVs over the past 14 years, with both showing peaks in the summer and troughs in the winter. RSVs for search terms that geolocate tattooing services have experienced a general increase in use since 2008. A compilation of results for all collated geolocating search terms localized these searches mainly to the American South, with lesser involvement in the eastern Midwest and inland West. Increased search interest in the Southeast at the expense of more populous coastal states and Great Plains or western Midwest states reflects the ongoing harmonization of tattoo prevalence across regions, as shown by national surveys. Searches for symptoms related to adverse reactions to tattooing experienced an increase over the period of interest, with the same distribution as previous global findings.

**Conclusions:**

Clinicians should be aware of an increase in search interest regarding tattoos and their removal, especially during the summer months in the Southeast and Midwest. This increase in interest is occurring together with increased tattoo prevalence and increased search interest for adverse reactions in a country lagging behind in tattoo ink regulation.

## Introduction

Given that an estimated 2% to 30% of tattoos are complicated by adverse effects [1], institutions in Europe have incrementally enacted recommendations and regulations for maximum allowed concentrations of various injurious compounds and elements in tattoo inks, beginning with a recommendation in 2008 by the Council of Europe, ResAP(2008)1 [2]. With comparatively little research on tattoos being conducted in the United States, and still less regulation, American clinicians are armed with less information with which to counsel and care for this patient population.

Research using Google Trends, a website that calculates relative search volumes (RSVs) of queries in representative samples of searches for specific time ranges and geographic regions [3], has shown steadily rising global search interest in tattoos [4] and their adverse effects [5]. However, such analysis has yet to be adapted specifically to the US, the world’s foremost producer of tattoo inks [6].

For this study, data were pulled from Google Trends, a website that calculates relative search volumes (RSVs) for queries in representative samples of searches for specific time ranges and geographic regions [7]. The data are freely available, anonymous, and unidentified. Queries are indexed as “topics” or “search terms”; topics include “a group of terms that share the same concept in any language” and so collate RSVs for multiple search terms [7]. Search interest was aggregated by topic to observe general trends over time, while isolating queries as search terms by subregion (ie, state) enabled observation of user attempts to geolocate tattooing services. Therefore, we sought to depict trends in search interest for tattoos across space and time.

## Methods

### Search Query Selection

To assess general interest in the application and removal of tattoo ink to and from the skin, respectively, the terms *tattoo*, indexed as a “visual art form,” and *tattoo removal*, indexed as a “topic,” were selected.

To observe trends in user geolocation of tattooing services, search terms specific to tattooing that localized the practice were collected from autocomplete results in the Google search engine, “top” and “rising” related queries in Google Trends, and other sources. Search strings with an RSV greater than one when compared to *tattoo shops*, the largest by volume, between January 15, 2008, and October 15, 2022, were tabulated (Table 1).

For each search string in Table 1, an average RSV was calculated from January 15, 2008, to October 15, 2015, and from October 15, 2015, to October 15, 2022. The proportions of these average RSVs to the total RSV for each of the 9 search terms were calculated for both the initial and final time periods as decimals.

Lastly, a comparison to prior research was made. Interest in the adverse effects of tattoos was modeled using symptomatic search terms adapted from Kluger [5]: *itchy tattoo*, *raised tattoo*, *swollen tattoo*, *tattoo bumps*, and *tattoo fading*. Trends in search interest were then compared to national surveys of tattoo prevalence conducted within the study’s timeframe.

**Table 1:** Google queries geolocating access to tattooing services and their relative search volumes.

Search string	Relative search volumes, % (percent in decimal)		
	Jan 15, 2008-Oct 15, 2022 (total)	Jan 15, 2008-Oct 15, 2015 (initial)	Oct 15, 2015-Oct 15, 2022 (final)
*tattoo shops*	31 (0.31)	62 (0.484)	46 (0.271)
*tattoo near me*	21 (0.21)	6 (0.0469)	42 (0.247)
*tattoo shop*	16 (0.16)	37 (0.289)	21 (0.124)
*tattoo shops near*	10 (0.1)	6 (0.0469)	20 (0.118)
*tattoo shops near me*	10 (0.1)	3 (0.0234)	19 (0.112)
*tattoos near me*	3 (0.03)	1 (0.00781)	7 (0.0412)
*tattoo parlor*	3 (0.03)	11 (0.0859)	3 (0.0177)
*tattoo shop near*	3 (0.03)	1 (0.00781)	6 (0.0353)
*tattoo shop near me*	3 (0.03)	1 (0.00781)	6 (0.0353)

### Search Characteristics

Google Trends was queried on October 23, 2022, with the selected topics and search terms. Results were filtered to web searches made in the United States. Results were not filtered by category, as the appropriate classification of searches as related to “arts & entertainment,” “health,” “shopping,” or other categories could not be independently verified.

### Data Retrieval and Analysis

RSVs for *tattoo* and *tattoo removal* were exported as a time series, with values being reported by Google Trends by month from January 15, 2008, to October 15, 2022. The data were imported into Prism 9 (version 9.3.1, GraphPad) to be visualized as a line graph.

RSVs for the geolocating search terms in Table 1 were exported by “subregion,” or state, for the periods January 15, 2008, to October 15, 2015, and October 15, 2015, to October 15, 2022. Both the initial and final RSVs for each search term were independently normalized by multiplication with the appropriate percent in decimal (Table 1). The normalized RSVs for the 2 time periods were then aggregated across the 9 search terms by state to give an initial and final relative search interest (RSI) for tattooing services. The pairs of RSIs were imported into the Datawrapper online app (Datawrapper GmBh) by state for visualization as choropleth maps of the US.

RSVs for the 5 selected symptomatic searches for adverse reactions to tattoos were packaged by month over the total time range of interest, from January 15, 2008, to October 15, 2022. Time-series data for each search term were exported from Google Trends and imported into Prism 9 for visualization. For clarity, the 5 sets of monthly data provided by Google Trends were averaged by year to produce line graphs depicting yearly mean RSVs with error bars representing the standard error of the mean.

## Results

### General Interest Searches

**Figure 1:**
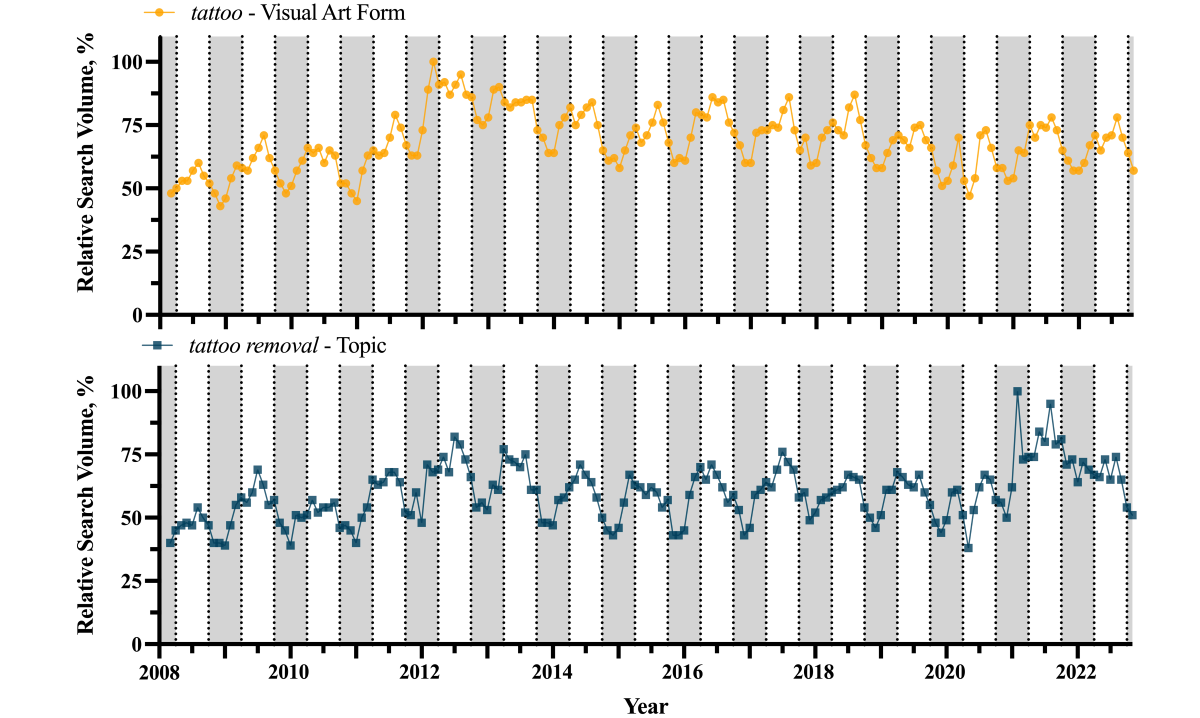
Relative search volumes for the Google Trends queries *tattoo*, indexed as a “visual art form,” and *tattoo removal*, indexed as a “topic.” Results were calculated by month from January 15, 2008, to October 15, 2022. Shaded areas denote time points for months from the beginning of September to the end of February.

The RSVs for *tattoo*, when indexed as a “visual art form,” and *tattoo removal*, when indexed as a “topic,” showed a cyclic wave pattern correlating with the seasons (Figure 1). Volume trends typically peaked in the spring and summer and reached a trough in the fall and winter. For *tattoo*, the month with the highest RSVs was typically July (11/14, 79%). The lowest monthly RSVs were most often reported for November (10/14, 71%). For *tattoo removal*, the month with the highest RSVs was most often June (5/14, 36%). The lowest monthly RSVs were most often reported for December (7/14, 50%).

### Searches Locating Access

**Figure 2:**
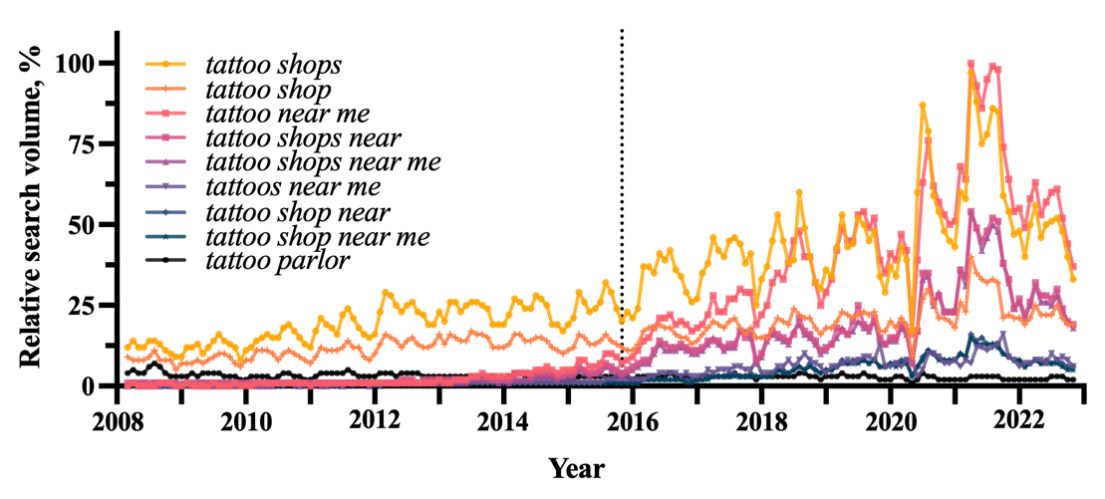
Relative search volumes for the search terms *tattoo shops*, *tattoo shop*, *tattoo near me*, *tattoo shops near*, *tattoo shops near me*, *tattoos near me*, *tattoo shop near*, *tattoo shop near me*, and *tattoo parlor* calculated by month from January 15, 2008, to October 15, 2022. The dotted line denotes October 2015.

Geolocating search terms with cumulative RSVs between January 15, 2008, and October 15, 2022, greater than one (Table 1) had their search interest plotted in Figure 2.

**Figure 3:**
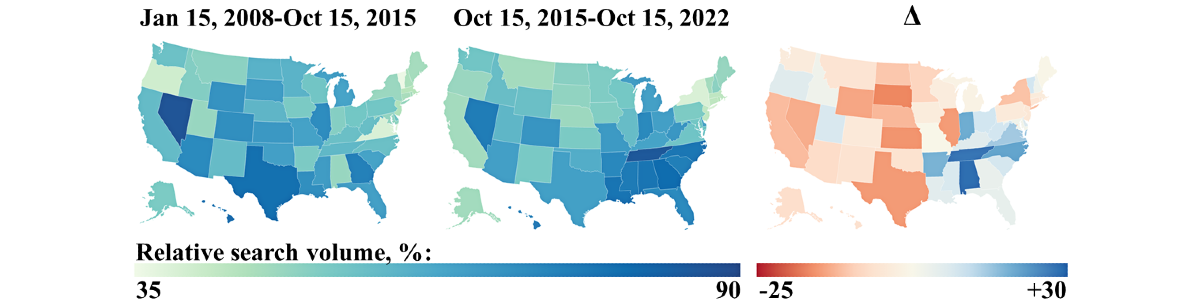
Relative search interest in tattoo service geolocation by state modeled by relative search volume (RSV). RSVs were calculated from January 15, 2008, to October 15, 2015 (left), and from October 15, 2015, to October 15, 2022 (center). The change in RSV by state, indicated by the Δ symbol (right), was calculated by subtracting the results of the initial time period from the results of the final time period.

Normalized aggregated RSVs, or RSIs, are visualized by state in Figure 3. The initial choropleth map revealed no pattern in RSI density with respect to population or geography, but the final map showed localization to the American South, extending into the Midwest, with lesser involvement in the inland West. This transition was driven by increases in RSI in the mid-South to the Midwest, paired with decreases in highly populated states and in the Great Plains. The largest increases in aggregate RSV over the time period of interest occurred in Alabama (29), Tennessee (28), and North Carolina (21). The largest decreases were seen in South Dakota (–16), Kansas (–15), and Texas (–14).

### Searches Based on Previous Work

**Figure 4:**
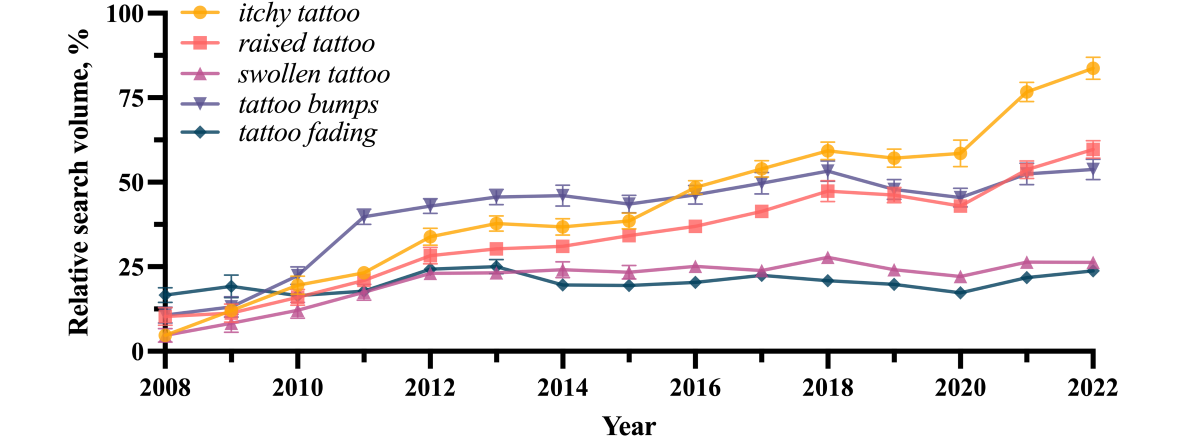
Relative search volumes for the search terms *itchy tattoo*, *raised tattoo*, *swollen tattoo*, *tattoo bumps*, and *tattoo fading* from January 15, 2008, to October 15, 2022, presented as yearly means. Errors bars represent the standard error of the mean.

The 5 search terms modeling Google web searches for symptoms of adverse reactions to tattoos have seen sustained or increased interest since January 15, 2008 (Figure 4). While yearly mean RSVs for *tattoo fading* and *swollen tattoo* were mostly stable between February 2008 and October 2022, *tattoo bumps* saw a sharp increase between 2008 and 2011, and *itchy tattoo* and *raised tattoo* have had sustained growth.

The results of freely available surveys assessing the regional prevalence of tattoos among Americans between 2008 and 2022 are tabulated in Table 2. While the West appears to initially predominate in terms of tattoo prevalence, there is a trend toward harmony among the 4 regions.

**Table 2:** Percentage of survey respondents with tattoos by region.

Surveyor (year)	Respondents by region, %			
	Northeast	Midwest	South	West
Harris (2008) [8]	12	10	13	20
Ipsos (2011) [9]	21	17	22	23
Harris (2012) [8]	21	21	18	26
Ipsos (2019) [10]	30	32	29	30

## Discussion

### Principal Results

Analysis of Google Trends data revealed a seasonal pattern in interest in tattooing and tattoo removal, with interest peaking in the summer and reaching a trough in the winter (Figure 1). Within this context, the use of the Google search engine to geolocate tattooing services has been exponentially increasing since at least 2015 (Figure 2), particularly in the Southeast (Figure 3). The localization of geolocation for tattoo services to the South, eastern Midwest, and inland West, regions considered more conservative, reflects an increase in popularity in perhaps more tattoo-naive areas, with consumers lacking industry knowledge. The decrease in RSI among western Midwest states and the Great Plains, however, defies this trend. Perhaps their distinctly rural character has so far delayed an uptick in tattoo popularity.

### Comparison With Prior Work

Increases in searches geolocating tattooing services in the South, as well as the Midwest, reflect the harmonization of tattoo prevalence across US regions, which has been noted by national surveys, although these have been scarce.

The US-derived results of this paper generally reflect findings from global data. Though general search interest for tattoos in the US has been relatively stable when compared to the sustained global increase, which has mainly been driven by Latin American countries [4] (Figure 1), the RSVs of the adapted symptom-related terms have stratified in the US in the same distribution as they have across the Anglosphere [5] (Figure 4).

### Limitations

Google Trends samples data generated from people who have internet access and use it, but these data are not necessarily reflective of these people’s behavior. Google Trends simply samples from a representative pool of queries within a timeframe and geographic region to generate relative data. Therefore, Google Trends results for a given timeframe and geographic region may slightly change with repeated sampling.

### Conclusions

Clinical dermatologists should be aware of the seasonal patterns associated with interest in tattoo application and removal and the effect of these patterns on tattoo care. Further research and surveillance are needed to understand the reasons behind and impact of geolocation of tattooing services, particularly in the American South in comparison to the Great Plains. Lastly, dermatologists should be aware of the interest in signs of localized inflammation when counseling patients on tattoo care and complications.

## Abbreviations

RSI: relative search interest
RSV: relative search volume
